# Achieving Enhanced Spectral Efficiency for Constant Envelope Transmission in CP-OFDMA Framework

**DOI:** 10.3390/s25237257

**Published:** 2025-11-28

**Authors:** Zhuhong Zhu, Yiming Zhu, Xiaodong Xu, Wenjin Wang, Li Chai, Yi Zheng

**Affiliations:** 1 National Mobile Communications Research Laboratory, Southeast University, Nanjing 210096, China; 220230836@seu.edu.cn (Z.Z.); ymzhu@seu.edu.cn (Y.Z.); 2 China Mobile Research Institute, Beijing 100032, China; chaili@chinamobile.com (L.C.); zhengyi@chinamobile.com (Y.Z.)

**Keywords:** constant-envelope, CP-OFDM, spectral efficiency, channel estimation, frequency-domain equalization

## Abstract

Orthogonal frequency-division multiplexing (OFDM) has been adopted as the baseline waveform for sixth-generation (6G) networks owing to its robustness and high spectral efficiency. However, its inherently high peak-to-average power ratio (PAPR) limits power amplifier efficiency and causes nonlinear distortion, particularly in power- and cost-constrained 6G scenarios. To address these challenges, we propose a constant-envelope cyclic-prefix OFDM (CE-CP-OFDM) transceiver under the CP-OFDMA framework, which achieves high spectral efficiency while maintaining low PAPR. Specifically, we introduce a spectrally efficient subcarrier mapping scheme with partial frequency overlap and establish a multiuser received signal model under frequency-selective fading channels. Subsequently, to minimize channel estimation error, we develop an optimal multiuser CE pilot design by exploiting frequency-domain phase shifts and generalized discrete Fourier transform-based time-domain sequences. For large-scale multiuser scenarios, a joint delay–frequency-domain channel estimation method is proposed, complemented by a low-complexity linear minimum mean square error (LMMSE) estimator in the delay domain. To mitigate inter-symbol and multiple-access interference, we further design an iterative frequency-domain LMMSE (FD-LMMSE) equalizer based on the multiuser joint received-signal model. Numerical results demonstrate that the proposed CE-CP-OFDM transceiver achieves superior bit-error-rate performance compared with conventional waveforms while maintaining high spectral efficiency.

## 1. Introduction

Orthogonal frequency-division multiplexing (OFDM) has become a fundamental modulation technique in modern wireless communication systems due to its robustness against frequency-selective fading, high spectral efficiency, and compatibility with multiple-antenna transmission [1,2]. Benefiting from these advantages, OFDM has also been adopted as the baseline waveform for sixth-generation (6G) mobile networks [3]. However, the inherently high peak-to-average power ratio (PAPR) of OFDM signals significantly limits the efficiency of power amplifiers (PAs) and introduces nonlinear distortion, thereby degrading overall system performance. This issue is particularly critical in power- and cost-constrained 6G scenarios such as low-power wide-area networks (LPWANs), unmanned aerial vehicle (UAV)-assisted networks, high-frequency terahertz communications, and non-terrestrial networks (NTNs) [3,4,5,6,7]. In this context, for the forthcoming 6G networks, it is essential to design new low-PAPR waveforms that preserve the intrinsic multicarrier structure of OFDM, thereby enabling high-reliability transmission with high data rates.

A wide range of schemes have been investigated to reduce the PAPR of OFDM signals, which can generally be grouped into three categories: signal distortion, probabilistic approaches, and coding-based methods [8,9]. Signal distortion techniques, such as clipping and filtering [10], peak windowing [11], companding [12], and peak cancellation [13], directly suppress the signal envelope but inevitably introduce in-band distortion and out-of-band radiation. Probabilistic approaches, including selective mapping (SLM) [14], partial transmit sequence (PTS) [15], and tone reservation (TR) [16], lower PAPR by generating alternative signal representations or assigning additional subcarriers for peak suppression, at the cost of side information, reduced rate, or higher complexity. Coding-based techniques, such as block codes [17] and Golay complementary sequences [18], can achieve simultaneous PAPR reduction and error correction, but are limited by coding rate constraints and scalability issues in broadband systems.

To overcome the limitations of conventional PAPR reduction techniques, the DFT-spread OFDM (DFT-s-OFDM) waveform was introduced in [19]. By applying a discrete Fourier transform (DFT) before inverse DFT (IDFT) modulation, DFT-s-OFDM effectively mitigates the PAPR problem of OFDM without significantly degrading bit-error-rate (BER) performance. However, its PAPR remains insufficiently low for high-efficiency power amplification. To further enhance power efficiency, constant-envelope OFDM (CE-OFDM) has been proposed, which transforms the amplitude-varying OFDM signal into a phase-modulated waveform to ensure the CE [20,21]. A fundamental challenge of CE-OFDM lies in the inherent trade-off between bit-error-rate (BER) performance and spectral efficiency. To overcome this challenge, CE-OFDM with index modulation (CE-OFDM-IM) integrates index modulation with CE-OFDM to exploit frequency diversity gains, thereby improving BER performance without consuming excessive bandwidth [22]. For the same purpose, CE-precoded OFDM (CE-P-OFDM) has been introduced to balance out-of-band leakage and BER performance [23]. Nevertheless, both schemes rely on phase modulation, which requires complicated phase-demodulation algorithms at the receiver, thereby hindering practical deployment [21]. In order to overcome the high receiver complexity associated with CE-OFDM, constant-amplitude OFDM (CA-OFDM) was proposed, which decomposes a time-domain OFDM signal into two constant-amplitude signals that are transmitted at different timings [24,25]. At the receiver, the original OFDM signal can be reconstructed simply by combining the two received signals, allowing subsequent processing to follow conventional OFDM procedures with low complexity. However, this approach incurs inherent drawbacks: the sequential transmission effectively doubles the required signal power, resulting in an intrinsic 3-dB performance loss, and the spectral efficiency is halved.

To overcome the high receiver complexity and BER performance degradation of the aforementioned CE schemes, we recently proposed a CE cyclic-prefix OFDM (CE-CP-OFDM) waveform under the CP orthogonal frequency-division multiple access (CP-OFDMA) framework [26,27,28]. This waveform employs a frequency-domain shaping filter to realize a CE single-carrier transmission within the CP-OFDM framework. The use of linear modulation enables low-complexity receiver processing while maintaining the CE property. However, as the design was tailored for scenarios with abundant bandwidth, it achieves only one-third of the spectral efficiency of conventional OFDM when ignoring the output back-off (OBO) of the PA. In addition, ref. [28] considers a multiuser scenario with orthogonal user allocation, effectively equivalent to a single-user system. As a result, the low-complexity receiver proposed in [28] is not suitable for scenarios with multiuser interference. Motivated by these limitations, this paper proposes a novel CE-CP-OFDM transceiver under the CP-OFDMA framework, which supports multiuser CE-CP-OFDMA with non-orthogonal subband overlap and achieves high spectral efficiency through multiuser-optimal CE pilot design, joint channel estimation, and equalization. The main contributions of this paper are summarized as follows:

- We propose a spectrally efficient subcarrier mapping scheme with partial inter-band overlap to prevent the spectral efficiency loss of CE-CP-OFDM. Furthermore, we establish the corresponding multiuser received signal model over frequency-selective fading channels.
- We propose an optimal multiuser CE pilot design that minimizes the joint channel estimation error in the delay domain. Specifically, we derive the frequency-domain pilot design condition that ensures minimal joint estimation error; i.e., the pilots are constructed as frequency-domain phase shifts to decouple different users in the delay domain. Based on this condition, we further construct a set of time-domain binary sequences under the CE constraint that satisfy the optimality criterion.
- To effectively mitigate multiuser pilot contamination that degrades channel estimation accuracy, we propose a joint delay–frequency-domain channel estimation scheme. In this scheme, users are first decoupled in the frequency domain, while remaining inseparable users are further decoupled in the delay domain using the property of phase-shift pilot. Moreover, a frequency-domain shift factor allocation strategy is designed to ensure sufficient decoupling among users. To further enhance the estimation performance, we also develop a low-complexity linear minimum mean square error (LMMSE) channel estimation method built upon the denoised least squares (LS) estimator in the delay domain.
- To combat inter-symbol interference (ISI) and multiple-access interference (MAI) in frequency-selective fading channels, a simplified received signal model for multiuser joint equalization is established, followed by an iterative frequency-domain LMMSE (FD-LMMSE) equalizer is designed to achieve efficient interference suppression.

The remainder of this paper is outlined as follows. Section 2 introduces the signal model of CE-CP-OFDM and presents a subcarrier mapping scheme with partial inter-band overlap. Section 3 develops optimal pilot design strategies under CE constraints, together with the corresponding channel estimation method. Section 4 describes an iterative FD-LMMSE-based equalizer. Section 5 provides simulation results and performance analysis. Finally, Section 6 concludes the paper.

Notations: Upper and lower case boldface letters denote matrices and column vectors, respectively. $I_{M}$ and $J_{M}$ are the M×M identity and reverse identity matrices, respectively. $0_{M\times N}$ and $1_{M\times N}$ denote all-zero and all-one matrices of size M×N, respectively. $C^{M\times N}$ and $R^{M\times N}$ represent complex and real matrix spaces of dimension M×N. $e_{k}$ is the *k*-th column of the identity matrix $I_{N_{c}}$. The superscripts ${\left(\cdot \right)}^{*}$, ${\left(\cdot \right)}^{T}$, ${\left(\cdot \right)}^{H}$ and ${\left(\cdot \right)}^{-1}$ denote conjugate, transpose, conjugate transpose and inverse, respectively. $\left|\right|\cdot {\left|\right|}_{F}$ and $\left|\right|\cdot {\left|\right|}_{2}$ denote the Frobenius norm and the $l_{2}$ norm, respectively. diag{d} denotes a diagonal matrix with d on the main diagonal. ${\left|d\right|}^{⊙2}$ denotes the element-wise squared modulus of d. ${\left[d\right]}_{m:n}$ denotes the subvector of d consisting of its elements from the *m*-th to the *n*-th. E{·} denotes statistical expectation. We denote ⊗ as the Kronecker product and d(m) as the *m*-th element of the column vector d. rank(A) and Tr(A) denote the rank and trace of matrix A, respectively. cov(x,y) is the covariance between x and y. Operators ⌈·⌉ and ⌊·⌋ stand for the ceiling and floor functions, respectively. ${\langle m\rangle }_{M}$ denotes *m* modulo *M*.

## 2. Transmitter Structure of the CE-CP-OFDMA Scheme

### 2.1. Signal Model

We consider an uplink offset quadrature amplitude modulation OFDM (OQAM-OFDM) system with *K* users, and the transmit signal of the *k*-th user is defined as [26,27]

(1) $$\chi_{D,k}\left(n\right)=\sum_{m=-\infty }^{+\infty }j^{k+m}d_{k}\left(m\right)g_{D,k}\left(n-m\Phi_{k}/2\right)e^{j2\pi f_{k}n},$$

where $d_{k}\left(m\right)$ is the *m*-th real-valued symbol obtained from the real and imaginary components of the QAM symbols, $f_{k}$ denotes the normalized center frequency, and $\Phi_{k}$ is the oversampling factor (typically $\Phi_{k}=K$). In (1), the transmit signal $\chi_{D,k}\left(n\right)$ is the superposition of an in-phase component and a quadrature component. Due to the OQAM structure, these two components have a $90^{\circ }$ phase difference and are staggered by $\Phi_{k}/2$ samples. When the input symbols $d_{k}\left(m\right)$ are constant-modulus (e.g., BPSK), one condition for achieving a constant-envelope transmit signal is that the pulse-shaping filter satisfies

(2) $$\left\{\begin{array}{cc} g_{D,k}^{2}\left(n\right)+g_{D,k}^{2}\;\left(\frac{\Phi_{k}}{2}-n\right)=1, & 0\leq |n|<\frac{\Phi_{k}}{2},\\ g_{D,k}^{2}\left(n\right)=0, & \left|n\right|\geq \frac{\Phi_{k}}{2}. \end{array}\right..$$

A practical sufficient condition to satisfy (2) is to use the cosine–sine–shaped pulse-shaping filter in [28]

(3) $$g_{D,k}\left(n\right)=\left\{\begin{array}{cc} 1, & n=0,\\ \cos\left(\Phi_{k}\left(n\right)\right), & 1\leq \left|n\right|\leq \frac{\Phi_{k}}{4}-1,\\ \frac{\sqrt{2}}{2}, & \left|n\right|=\frac{\Phi_{k}}{4},\\ \sin\left(\Phi_{k}\left(\frac{\Phi_{k}}{2}-n\right)\right), & \frac{\Phi_{k}}{4}+1\leq \left|n\right|\leq \frac{\Phi_{k}}{2}-1,\\ 0, & \mathrm{otherwise}, \end{array}\right.$$

With this pulse, the resulting transmit signal of the *k*-th user exhibits the CE property.

To reduce the equalization complexity of OQAM-OFDM, the CP-OQAM-OFDM system introduces a CP, which converts the linear convolution with the multipath channel into a circular one and thereby enables low-complexity frequency-domain equalization. In this system, user data symbols are first organized into blocks, i.e., OFDM symbols, with a CP inserted between adjacent blocks to mitigate inter-block interference. For the *k*-th user, each block is assumed to consist of $N_{d,k}$ QAM symbols, resulting in $N_{c}=N_{d,k}\Phi_{k}$ time-domain samples per block. Based on this assumption, the transmit signal of the *l*-th block for the *k*-th user, prior to CP insertion, can be expressed as

(4) $$\chi_{k,l}\left(n\right)=\sum_{m=0}^{2N_{d,k}-1}j^{k+m}e^{j\pi f_{k}m\Phi_{k}}d_{k,l}\left(m\right)g_{k}\left({\langle n-m\Phi_{k}/2\rangle }_{N_{c}}\right),\;n\in \left\{0,1,\ldots ,N_{c}-1\right\}$$

where the filter $g_{k}\left(n\right)$ for the *k*-th user is defined as [27]

(5) $$g_{k}\left(n\right)=\left\{\begin{array}{cc} {\bar{g}}_{k}\left(n\right), & 0\leq n\leq \frac{\Phi_{k}}{2}-1,\\ {\bar{g}}_{k}\left(n-N_{c}+1\right), & N_{c}-\frac{\Phi_{k}}{2}\leq n\leq N_{c}-1,\\ 0, & \mathrm{otherwise}, \end{array}\right.$$

with ${\bar{g}}_{k}\left(n\right)=g_{d,k}\left(n\right)e^{j2\pi f_{k}n},\;1-\Phi_{k}/2\leq n\leq \Phi_{k}/2-1$.

The transmit signal in (4) can be expressed in a vector form $\chi_{k,l}≜[\chi_{k,l}\left(0\right),\chi_{k,l}\left(1\right),\cdots ,$$\chi_{k,l}\left(N_{c}-1\right){]}^{T}$ as

(6) $$\chi_{k,l}=j^{k}{\tilde{G}}_{k}M_{k}\Omega_{k}d_{k,l},$$

where ${\tilde{G}}_{k}=\mathrm{circ}\left(g_{k}\right)$ denotes a circulant matrix generated from $g_{k}=[g_{k}\left(0\right),g_{k}\left(1\right),\cdots$, $g_{k}\left(N_{c}-1\right){]}^{T}$. The matrix $M_{k}=\left[e_{0},e_{\Phi_{k}/2},\ldots ,e_{\left(2N_{d,k}-1\right)\Phi_{k}/2}\right]$, where $e_{i}$ denotes the *i*-th column of the $N_{c}\times N_{c}$ identity matrix, serves as a zero-padding interpolation matrix with nonzero entries spaced by $\Phi_{k}/2$. The real-valued data vector $d_{k,l}≜{\left[d_{k,l}\left(0\right),d_{k,l}\left(1\right),\cdots ,d_{k,l}\left(2N_{d,k}-1\right)\right]}^{T}$. The diagonal matrix $\Omega_{k}=\mathrm{diag}([1,e^{j\pi (f_{k}\Phi_{k}+0.5)},\ldots$, $e^{j\pi \left(2N_{d,k}-1\right)\left(f_{k}\Phi_{k}+0.5\right)}])$ applies frequency modulation to the input symbols. To facilitate the implementation and optimal design of the CP-OQAM-OFDM system, we define the normalized center frequency as $f_{k}=\frac{a_{k}^{\prime }-\left\lfloor \left(N_{d,k}+1\right)/2\right\rfloor -\delta_{k}}{N_{c}}$, where $a_{k}^{\prime }$ is an integer index and $\delta_{k}$ equals 0.5 and 0 for even and odd $N_{d,k}$, respectively. Under this definition, the diagonal matrix $\Omega_{k}$ can be equivalently expressed as $\Omega_{k}=\mathrm{diag}\left(\left[1,e^{j\pi \frac{a_{k}^{\prime }}{N_{d,k}}},\ldots ,e^{j\pi \frac{\left(2N_{d,k}-1\right)a_{k}^{\prime }}{N_{d,k}}}\right]\right)\cdot \Phi_{2N_{d,k}}$, where $\Phi_{2N_{d,k}}$ is a $2N_{d,k}\times 2N_{d,k}$ diagonal matrix with its *n*-th diagonal entry given by $e^{-j\pi n/(2N_{d,k})}$.

Equation (6) describes the time-domain filtering structure of the transmit signal. To enable efficient implementation in the CP-OFDM system, it is necessary to reformulate (6) into an equivalent frequency-domain filtering structure. Since ${\tilde{G}}_{k}$ is a circulant matrix, the corresponding eigenvalue decomposition is given by [29]${\tilde{G}}_{k}=W_{N_{c}}^{H}\Lambda_{k}W_{N_{c}}$, where $W_{N_{c}}$ denotes the $N_{c}\times N_{c}$ normalized DFT matrix with its (m,n)-th entry given by ${\left[W_{N_{c}}\right]}_{m,n}=\frac{1}{\sqrt{N_{c}}}e^{-j\frac{2\pi mn}{N_{c}}}$. The matrix $\Lambda_{k}=\mathrm{diag}\left(\lambda_{k}\right)$ is a diagonal matrix, where $\lambda_{k}=\sqrt{N_{c}}W_{N_{c}}g_{k}$. Therefore, (6) can be further written as

(7) $$\chi_{k,l}=j^{k}W_{N_{c}}^{H}\Lambda_{k}W_{N_{c}}M_{k}\Omega_{k}d_{k,l}.$$

From (7), the structure can be interpreted as a frequency-domain filtering operation, which is equivalent to applying zero-padding in the time domain followed by an $N_{c}$-point DFT. It is worth noting that this operation is equivalent to performing a $2N_{d,k}$-point DFT combined with frequency-domain periodic extension, while the latter leads to a lower implementation complexity. Consequently, the filtering of the transmit signal in (6) can be equivalently reformulated in the frequency domain as [27]

(8) $$\chi_{k,l}=W_{N_{c}}^{H}P_{k}{\tilde{W}}_{2N_{d,k}}d_{k,l},$$

where $P_{k}=j^{k}\sqrt{\frac{2}{\Phi_{k}}}\Lambda_{k}E_{2N_{d,k}}T_{k}\in C^{N_{c}\times 2N_{d,k}}$ represents the frequency-domain transmit processing (FDTP) matrix for the *k*-th user. Here, $E_{2N_{d,k}}=1_{\Phi_{k}/2\times 1}⊗I_{2N_{d,k}}$ is the frequency-domain periodic extension matrix, which corresponds to a zero-padding interpolation operation in the time domain. The frequency-domain circular shift matrix $T_{k}$ is defined as

(9) $$T_{k}=\left[\begin{array}{cc} 0 & I_{{\langle a_{k}^{\prime }\rangle }_{2N_{d,k}}}\\ I_{2N_{d,k}-{\langle a_{k}^{\prime }\rangle }_{2N_{d,k}}} & 0 \end{array}\right],$$

which is equivalent to a frequency modulation operation in the time domain. Moreover, ${\tilde{W}}_{2N_{d,k}}=W_{2N_{d,k}}\Phi_{2N_{d,k}}$ is the $2N_{d,k}$-point generalized DFT (GDFT) matrix, which transforms the real-valued input vector $d_{k,l}$ into a $2N_{d,k}$-point frequency-domain vector $q_{k,l}={\tilde{W}}_{2N_{d,k}}d_{k,l}$ exhibiting exact conjugate symmetry, i.e., $q_{k,l}\left(m\right)=q_{k,l}^{*}\left(2N_{d,k}-1-m\right)$, $0\leq m<2N_{d,k}$. Thus, $q_{k,l}$ can be compactly expressed as

(10) $$q_{k,l}={\left[\begin{array}{cc} s_{k,l}^{T} & {\left(J_{N_{d,k}}s_{k,l}^{*}\right)}^{T} \end{array}\right]}^{T},$$

where $s_{k,l}={\left[q_{k,l}\left(0\right),\ldots ,q_{k,l}\left(N_{d,k}-1\right)\right]}^{T}\in C^{N_{d,k}\times 1}$ denotes the frequency-domain data vector of the *k*-th user.

To ensure the compatibility with DFT-s-OFDM and avoid the computational redundancy of applying a $2N_{d,k}$-point real-valued GDFT as in (10), the CE-CP-OFDM system adopts an optimized structure based on an $N_{d,k}$-point complex-valued GDFT [28]. Specifically, the frequency-domain data $q_{k,l}\in C^{2N_{d,k}\times 1}$ is expressed as

(11) $$q_{k,l}=\frac{1}{2\sqrt{2}}\left[\begin{array}{cc} I_{N_{d,k}} & I_{N_{d,k}}\\ I_{N_{d,k}} & -I_{N_{d,k}} \end{array}\right]\left[\begin{array}{cc} I_{N_{d,k}} & 0\\ 0 & -je^{-j\pi /(2N_{d,k})}\Phi_{N_{d,k}} \end{array}\right]\left[\begin{array}{cc} I_{N_{d,k}} & J_{N_{d,k}}\\ I_{N_{d,k}} & -J_{N_{d,k}} \end{array}\right]\left[\begin{array}{c} {\bar{q}}_{k,l}\\ {\left({\bar{q}}_{k,l}\right)}^{*} \end{array}\right],$$

where

(12) $${\bar{q}}_{k,l}=W_{N_{d,k}}\Phi_{N_{d,k}}{\bar{d}}_{k,l}\in C^{N_{d,k}\times 1}.$$

The complex-valued quadrature PSK (QPSK) symbol vector is defined as ${\bar{d}}_{k,l}={\left[{\bar{d}}_{k,l}\left(0\right),{\bar{d}}_{k,l}\left(1\right),\cdots ,{\bar{d}}_{k,l}\left(N_{d,k}-1\right)\right]}^{T}$, with ${\bar{d}}_{k,l}\left(m\right)=d_{k,l}\left(2m\right)+jd_{k,l}\left(2m+1\right)$.

### 2.2. Subcarrier Allocation

**Figure 1:**
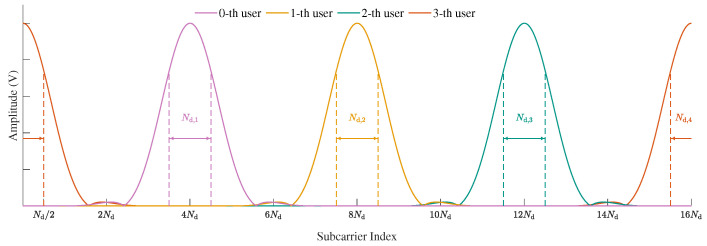

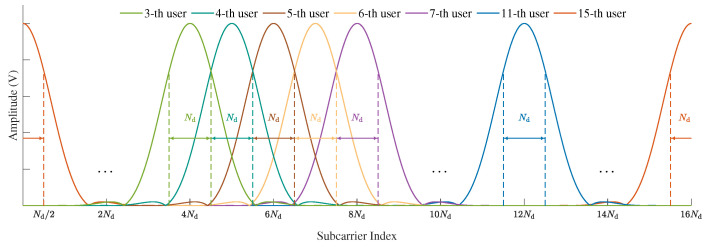
Frequency domain waveform diagram of the proposed CE-CP-OFDMA with sufficient guard interval (**above**) and without a guard interval (**below**) between adjacent users.

To mitigate MAI caused by the excessive bandwidth of CE waveform filters, the method in [28] adopts the subcarrier mapping strategy illustrated above in Figure 1, where each user is allocated sufficient bandwidth. For example, the subband allocated to the *k*-th user spans approximately $3N_{d,k}$ OFDM subcarriers for the transmission of $N_{d,k}$ QPSK symbols, thereby nearly eliminating multiuser MAI at the cost of reduced spectral efficiency. To enable CE waveform transmission without compromising spectral efficiency, we propose a spectrally efficient subcarrier allocation strategy that allows partial subcarrier overlap among adjacent users. For simplicity, we assume that all users are allocated the same bandwidth, i.e., $N_{d,0}=\cdots =N_{d,K-1}=N_{d}$, and therefore adopt identical sampling factors, i.e., $\Phi_{0}=\cdots =\Phi_{K-1}=\Phi =N_{c}/N_{d}$. As illustrated below in Figure 1, the center-frequency spacing between adjacent users is set to $N_{d}$ subcarriers, where the central subcarrier index of the *k*-th user is given by $a_{k}=a+kN_{d}$, with a∈Z representing the starting subcarrier index of the 0-th user in the system. Such a subcarrier allocation scheme preserves the CE property while achieving the same spectral efficiency as conventional QPSK-modulated OFDMA. Specifically, each user can transmit $N_{d}$ QPSK data symbols over a subband spanning $N_{d}$ subcarriers in one OFDM symbol. Nevertheless, the introduced subcarrier overlap results in severe inter-user interference, which significantly degrades both channel estimation and signal detection performance at the receiver. To address these challenges, in the following, we develop a low-complexity channel estimation method tailored for this partially overlapping subcarrier scenario, followed by a frequency-domain LMMSE detection algorithm for mitigating the residual interference.

## 3. Pilot Design and Channel Estimation

### 3.1. Received Signal Model

We consider an uplink CE-CP-OFDMA system with *K* users, where each user experiences a frequency-selective fading channel due to multipath propagation. For the *k*-th user in the *l*-th transmission block, the channel impulse response is modeled as a superposition of $P_{k,l}$ discrete propagation paths [30]

(13) $${\bar{h}}_{k,l}^{\tau }\left(\tau \right)=\sum_{p=0}^{P_{k,l}-1}\alpha_{k,l,p}\;\delta \left(\tau -\tau_{k,l,p}\right),$$

where $\alpha_{k,l,p}$ and $\tau_{k,l,p}\in \left[0,\frac{L}{N_{c}\Delta f}\right]$ denote the complex gain and delay of the *p*-th path. Here, *L* denotes the maximum number of resolvable channel taps among all users and Δf denotes the system subcarrier spacing. The path gain $\alpha_{k,l,p}$ incorporates both large-scale and small-scale fading effects and is given by $\alpha_{k,l,p}=\sqrt{\beta_{k}}\cdot \zeta_{k,l,p}$, where $\beta_{k}$ denotes the path loss and shadowing component of the *k*-th user, and $\zeta_{k,l,p}∼\mathrm{CN}\left(0,\sigma_{\tau ,k,l,p}^{2}\right)$ represents the independent and non-identically distributed (i.n.d.) Rayleigh fading component, where $\sigma_{\tau ,k,l,p}^{2}$ denotes the average power of the *p*-th path between the base station and the *k*-th user in the *l*-th transmission block. The parameters ${\left\{\sigma_{\tau ,k,l,p},\tau_{,k,l,p}\right\}}_{p=0}^{P_{k,l}-1}$ are drawn based on the power delay profile (PDP), which specifies the average power and delay distribution of multipath components. δ(·) is the Dirac delta function.

We obtain the frequency-domain channel response over $N_{c}$ subcarriers by performing the Fourier transform of the channel impulse response at the subcarrier frequencies ${\left\{f_{n}=n\Delta f\right\}}_{n=0}^{N_{c}-1}$. The resulting frequency-domain channel vector ${\bar{h}}_{k,l}\in C^{N_{c}\times 1}$ is expressed as

(14) $${\bar{h}}_{k,l}=\sum_{p=0}^{P_{k,l}-1}\alpha_{k,l,p}\;b_{N_{c}}\left(\tau_{k,l,p}\right),$$

where the delay-domain steering vector $b_{N_{c}}\left(\tau \right)={\left[\;1,\;e^{-j2\pi \Delta f\tau },\;\ldots ,\;e^{-j2\pi (N_{c}-1)\Delta f\tau }\;\right]}^{T}$.

**Figure 2:**
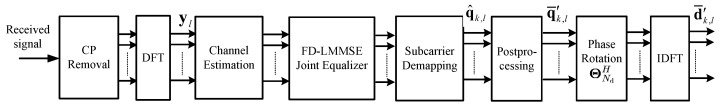
The receiver structure of the proposed CE-CP-OFDM within the framework of CP-OFDMA.

As illustrated in Figure 2, the CP is first removed and the DFT is performed at the receiver. The resulting frequency-domain received signal $y_{l}\in C^{Nc\times 1}$ in the *l*-th block, over frequency-selective fading channels can then be expressed, based on (8) and (14), as

(15) $$y_{l}=\sqrt{\frac{2}{\Phi }}\sum_{k=0}^{K-1}j^{k}\mathrm{diag}\left({\bar{h}}_{k,l}\right)\Lambda_{k}E_{2N_{d}}T_{k}q_{k,l}+z_{l}={\bar{X}}_{l}{\bar{\xi }}_{l}+z_{l},$$

where the aggregated equivalent pilot matirx ${\bar{X}}_{l}=\left[{\bar{X}}_{0,l},\;{\bar{X}}_{1,l},\;\ldots ,\;{\bar{X}}_{K-1,l}\right]$ with ${\bar{X}}_{k,l}=\mathrm{diag}\left({\bar{x}}_{k,l}\right)$. ${\bar{x}}_{k,l}=j^{k}\sqrt{2/\Phi }\;E_{2N_{d}}T_{k}q_{k,l}$ is the corresponding equivalent frequency-domain pilot vector of the *u*-th user. The aggregated equivalent channel vector ${\bar{\xi }}_{l}=[{\bar{\xi }}_{0,l}^{T},{\bar{\xi }}_{1,l}^{T},\ldots ,$${\bar{\xi }}_{K-1,l}^{T}{]}^{T}$, with ${\bar{\xi }}_{k,l}=\Lambda_{k}{\bar{h}}_{k,l}$ denoting the equivalent frequency-domain channel of the *u*-th user. The noise term $z_{l}$ is modeled as an additive white Gaussian noise (AWGN) vector with zero mean and covariance $\sigma_{z}^{2}I_{N_{c}}$. For notational simplicity, the block index *l* will be omitted in the subsequent analysis unless otherwise specified.

### 3.2. Pilot Sequence Design Under CE Constraints

According to the received signal model in (15), the dimension of the observation vector is $N_{c}$, while the total dimension of the unknown frequency-domain channel parameters across all users is $KN_{c}$. Owing to such dimensionality mismatch, the frequency-domain channel estimation problem is underdetermined and thus infeasible to solve directly. Considering the high correlation of frequency-domain channel responses across subcarriers, the eigenvector matrix can be employed to decorrelate the frequency-domain channel response vector to the sparsity-domain one, thereby substantially reducing the estimation dimensionality. Accordingly, the pilot received signal model in (15) is reformulated as

(16) $$\begin{array}{cc} y & =\sum_{k=0}^{K-1}{\bar{X}}_{k}W_{N_{c}}P^{T}{\bar{\xi }}_{k}^{\tau }+z=S{\bar{\xi }}^{\tau }+z, \end{array}$$

where the delay-domain equivalent channel vector of the *k*-th user is defined as ${\bar{\xi }}_{k}^{\tau }=PW_{N_{c}}^{H}{\bar{\xi }}_{k}\in C^{(L+1)\times 1}$, and $P=\left[I_{L+1},\;0_{\left(L+1\right)\times \left(N_{c}-L-1\right)}\right]\in R^{\left(L+1\right)\times N_{c}}$ is a selection matrix that extracts the first (L+1) delay taps. The equivalent pilot matrix is $S=\left[{\bar{X}}_{0}W_{N_{c}}P^{T},\;{\bar{X}}_{1}W_{N_{c}}P^{T},\;\ldots ,\;{\bar{X}}_{K-1}W_{N_{c}}P^{T}\right]\in C^{N_{c}\times K\left(L+1\right)}$, and the composite delay-domain channel vector is ${\bar{\xi }}^{\tau }={\left[\;{\bar{\xi }}_{0}^{T},\;{\bar{\xi }}_{1}^{T},\;\ldots ,\;{\bar{\xi }}_{K-1}^{T}\;\right]}^{T}\in C^{K(L+1)\times 1}$.

Given that the channel length *L* is generally smaller than the CP length $N_{g}$, the condition $N_{c}\geq K\left(L+1\right)$ can be practically ensured through appropriate user scheduling and resource allocation. Consequently, the LS estimate of ${\bar{\xi }}^{\tau }$ is given by

(17) $${\bar{\xi }}_{\mathrm{ls}}^{\tau }={\left(S^{H}S\right)}^{-1}S^{H}y\in C^{K(L+1)\times 1}.$$

Considering that $S^{H}S$ is a K(L+1)×K(L+1) matrix, the computational complexity of the matrix inversion in the LS estimator grows significantly as either *K* or *L* increases. To reduce complexity, it is desirable for the pilot matrix S to satisfy the orthogonality condition

(18) $$S^{H}S=cI_{K(L+1)},$$

where *c* denotes the average power of the pilot signal. This condition eliminates the need for matrix inversion in the LS estimator and ensures the minimum mean squared error (MSE) performance [31]. It can be shown that (18) is satisfied if the frequency-domain pilot matrices of different users fulfill

(19) $$\begin{array}{c} B_{mn}=PW_{N_{c}}^{H}{\bar{X}}_{m}^{H}{\bar{X}}_{n}W_{N_{c}}P^{T}=c\delta \left[m-n\right]I_{L+1},\;m,n\in \left\{0,1,\ldots ,K-1\right\}, \end{array}$$

where δ[·] is the Kronecker delta function.

According to (19), the matrix $W_{N_{c}}P^{T}$ extracts the first (L+1) orthonormal DFT basis vectors, whereas $PW_{N_{c}}^{H}$ is simply the conjugate transpose of this set of basis vectors. Accordingly, the matrix ${\bar{X}}_{m}^{H}{\bar{X}}_{n}W_{N_{c}}P^{T}$ can be interpreted as a new set of basis vectors obtained by applying the linear transformation ${\bar{X}}_{m}^{H}{\bar{X}}_{n}W_{N_{c}}$ to these (L+1) orthonormal DFT basis vectors. Thus, the matrix $PW_{N_{c}}^{H}{\bar{X}}_{m}^{H}{\bar{X}}_{n}W_{N_{c}}P^{T}$ may be viewed as the inner-product matrix between the two resulting sets of basis vectors, and the orthogonality condition in (19) is satisfied whenever every pair of basis vectors across the two sets is mutually orthogonal, i.e., $B_{mn}=0$ for all m≠n. Since any two non-overlapping subsets of DFT basis vectors in $W_{N_{c}}$ are inherently orthogonal and can be mapped to one another through an appropriate linear phase-shift matrix, if the frequency-domain pilots ${\bar{X}}_{m}$ and ${\bar{X}}_{n}$ differ by a properly designed linear phase term, then ${\bar{X}}_{m}^{H}{\bar{X}}_{n}W_{N_{c}}P^{T}$ can be shifted into a DFT subspace that is disjoint from the one associated with $W_{N_{c}}P^{T}$. Because these two sets of basis vectors are then non-overlapping and hence orthogonal, the orthogonality condition in (19) is guaranteed to hold.

Motivated by this intuition, we next examine how to design phase-shifted pilots under the CE structure. Expanding (19) yields $B_{mn}=PW_{N_{c}}^{H}{\left(I_{\Phi /2}⊗X_{m}\right)}^{H}\left(I_{\Phi /2}⊗X_{n}\right)W_{N_{c}}P^{T}$. Here, $X_{k}=\mathrm{diag}\left(x_{k}\right)\in C^{2N_{d}\times 2N_{d}}$, and $x_{k}=j^{k}\sqrt{2/\Phi }\;T_{k}q_{k}\in C^{2N_{d}\times 1}$ denotes the frequency-domain pilot of the *k*-th user prior to periodic extension. Following the phase-shift structure, we construct $x_{k}=e^{j\theta_{k}}\Theta_{k}x_{0}$, where $e^{j\theta_{k}}$ is an arbitrary initial phase offset, and $\Theta_{k}$ is a diagonal phase-rotation matrix defined as

(20) $$\Theta_{k}=\mathrm{diag}\left(1,e^{-j\frac{2\pi \Delta_{k}}{2N_{d}}},\ldots ,e^{-j\frac{2\pi \Delta_{k}\left(2N_{d}-1\right)}{2N_{d}}}\right)\in C^{2N_{d}\times 2N_{d}},$$

and $\Delta_{k}$ denotes the frequency-domain shift factor of the *k*-th user. The (u,v)-th element of the matrix $B_{mn}$ can be expressed as

(21) $$\begin{array}{cc} {\left[B_{mn}\right]}_{u,v} & =\sum_{\phi =0}^{\phi =\Phi /2-1}\sum_{k=0}^{2N_{d}-1}x_{m}^{*}\left(k\right)x_{n}\left(k\right)e^{-j\frac{2\pi \left(v-u\right)\left(k+2\phi N_{d}\right)}{N_{c}}}=ce^{j(\theta_{n}-\theta_{m})}\frac{1-e^{j\pi \left(2u-2v+\Phi \left(\Delta_{m}-\Delta_{n}\right)\right)}}{1-e^{j\frac{2\pi \left(u-v+\Phi /2\left(\Delta_{m}-\Delta_{n}\right)\right)}{N_{c}}}}, \end{array}$$

where u,v∈{0,1,…,L}. From (21), we observe that $B_{mn}=c\;I_{L+1}$ when m=n. Moreover, for m≠n, if ${\langle u-vs.+\frac{\Phi }{2}\left(\Delta_{m}-\Delta_{n}\right)\rangle }_{N_{c}}\neq 0$, then $B_{mn}=0_{L+1}$. Without loss of generality, the phase-shifting factor is assumed to satisfy $\Delta_{m},\Delta_{n}\in \left\{0,1,\ldots ,2N_{d}\right\}$. Under this condition, we have the following proposition.

**Proposition** **1.** *In the CE-CP-OFDM system, the frequency-domain pilot matrices of different users satisfy the optimal orthogonality condition in *(18)* if they meet the conditions below:*

(22) $$\left\{\begin{array}{c} X_{m}=e^{j\theta_{m}}\Theta_{m}X_{0},\\ X_{0}^{H}X_{0}=cI_{2N_{d}},\\ \frac{2L}{\Phi }<\left|\Delta_{m}-\Delta_{n}\right|<2N_{d},\;m\neq n,\;m,n\in \left\{0,1,\ldots ,K-1\right\} \end{array}\right.$$

Proposition 1 states the conditions for optimal frequency-domain pilots. However, in the CE-CP-OFDM system, the time-domain pilots are constrained to binary sequences, thereby necessitating the design of frequency-domain pilots that satisfy Proposition 1 under this binary constraint. As Reference [28] has thoroughly described how to design $x_{0}$ based on the binary sequence $d_{0}$ such that $X_{0}^{H}X_{0}\approx cI_{2N_{d}}$ within the GDFT framework, the remaining challenge is to develop frequency-domain phase-shifted pilots under the same constraint within the GDFT framework. Based on the relationship between $x_{m}$ and $q_{m}$, the desired frequency-domain phase-shifted pilots can be constructed if $q_{m}$ and $q_{0}$ satisfy the following condition:

(23) $$\begin{array}{cc} q_{m} & =j^{-m}e^{j\theta_{m}}T_{m}^{-1}\Theta_{m}T_{0}q_{0}. \end{array}$$

According to the properties of the circular shift matrix defined in (9), the expression in (23) can be further rewritten as

(24) $$\begin{array}{cc} q_{m} & =j^{-m}e^{j\theta_{m}}\left[\begin{array}{cc} 0 & I_{{\langle mN_{d}\rangle }_{2N_{d}}}\\ I_{{\langle mN_{d}\rangle }_{2N_{d}}} & 0 \end{array}\right]T_{0}^{-1}\Theta_{m}T_{0}q_{0}=j^{-m}e^{j\theta_{m}}e^{-j\pi a/N_{d}}{\bar{T}}_{N_{d}}^{m}\Theta_{m}q_{0}, \end{array}$$

where a∈Z represents the starting subcarrier index of the 0-th user in the system, and ${\bar{T}}_{N_{d}}$ is a cyclic shift matrix that circularly shifts a length-$2N_{d}$ vector by $N_{d}$ defined as

(25) $$\begin{array}{c} {\bar{T}}_{N_{d}}=\left[\begin{array}{cc} 0 & I_{N_{d}}\\ I_{N_{d}} & 0 \end{array}\right]. \end{array}$$

After obtaining (24), we further investigate the relationship between the binary sequences $d_{m}$ and $d_{0}$. Based on the relationship between $d_{m}$ and $q_{m}$, we can derive

(26) $$\begin{array}{cc} d_{m} & ={\tilde{W}}_{2N_{d,k}}^{H}j^{-m}e^{j\theta_{m}}e^{-j\pi a/N_{d}}{\bar{T}}_{N_{d}}^{m}\Theta_{m}q_{0}=e^{j\theta_{m}}e^{-j\pi \left(2a+mN_{d}-\Delta_{m}\right)/2N_{d}}D^{m}{\tilde{T}}_{m}d_{0}, \end{array}$$

where $D\in R^{2N_{d}\times 2N_{d}}$ is a diagonal matrix whose (m,m)-th entry is given by $D\left(m,m\right)={\left(-1\right)}^{m}$ for $m\in \{0,1,\ldots ,2N_{d}-1\}$, and ${\tilde{T}}_{m}\in R^{2N_{d}\times 2N_{d}}$ is a circular-shift transformation matrix defined as

(27) $${\tilde{T}}_{m}=\left[\begin{array}{cc} 0 & -I_{\Delta_{m}}\\ I_{2N_{d}-\Delta_{m}} & 0 \end{array}\right].$$

Based on (27), it can be observed that both transformation matrices $D^{m}$ and ${\tilde{T}}_{m}$ ensure that the output sequence remains binary. Therefore, when $\theta_{m}=\pi \left(mN_{d}+2a-\Delta_{m}\right)/\left(2N_{d}\right)$, a set of time-domain binary pilot sequences can be constructed according to (26), whose corresponding frequency-domain pilots exhibit the desired phase-shifted structure. As derived above, the procedure for generating the optimal time-domain pilot sequences for multiuser transmission in the CE-CP-OFDM system is summarized in Algorithm 1.
**Algorithm 1** Multiuser Optimal Pilot Design Algorithm**Input: **$N_{d},K,\Phi ,L$1:Generate the prototype sequence $d_{0}\in R^{2N_{d}\times 1}$ using the CE-constrained pilot optimization algorithm proposed in [28]2:Set the phase shift parameter Δ such that $2L/\Phi <\Delta <2N_{d}/K$3:**for** k=1 to K−1 **do**4:    Construct $d_{k}\left(n\right)=d_{0}\left({\langle n-k\Delta \rangle }_{2N_{d}}\right),\;n\in \left\{1,2,\ldots ,2N_{d}\right\}$5:    Multiply the first kΔ elements of $d_{k}$ by −16:    **if** *k* is odd **then**7:        **for** m=1 to $N_{d}$ **do**8:           $d_{k}\left(2m\right)=-d_{k}\left(2m\right)$9:        **end for**10:    **end if**11:**end for****Output:** Binary pilot sequences ${\left\{d_{k}\right\}}_{k=0}^{K-1}$

Based on the designed phase-shifted pilot sequences and (17), the delay-domain equivalent channel vector for the *k*-th user can be readily obtained as ${\bar{\xi }}_{k,\mathrm{dl}}^{\tau }=\frac{1}{c}PW_{N_{c}}^{H}{\bar{X}}_{k}^{H}y\in C^{(L+1)\times 1}$. Then, the corresponding estimate of the frequency-domain equivalent channel for the *k*-th user is expressed as

(28) $${\bar{\xi }}_{k,\mathrm{dl}}=\frac{1}{c}W_{N_{c}}P^{T}PW_{N_{c}}^{H}{\bar{X}}_{k}^{H}y\in C^{N_{c}\times 1}.$$

By analyzing (28), it can be observed that this operation essentially performs user separation in the delay domain using phase-shifted pilots, followed by noise suppression using a rectangular window. By assuming that the actual multipath delay spread of each user lies entirely within the CP duration and that the frequency-domain pilots satisfy the optimal phase-shift structure in (22), the channel estimation performance is affected only by measurement noise, while specification and sampling errors can be neglected. Under this condition, the resulting channel estimation error variance is

(29) $$\sigma_{\mathrm{dl}}^{2}=\sigma^{2}\mathrm{Tr}\;\left({\left(S^{H}S\right)}^{-1}\right)=\frac{K(L+1)}{c}\;\sigma_{z}^{2}.$$

### 3.3. Joint Delay-Frequency Domain Channel Estimation

The phase-shifted pilot design proposed in the previous subsection facilitates multiuser channel decoupling in the delay domain, thereby improving channel estimation accuracy by mitigating interference from overlapping pilot signals. However, as illustrated in Algorithm 1, the construction of mutually orthogonal phase-shifted pilots becomes challenging when the number of users *K* or the maximum channel taps *L* is large. In such cases, it may be infeasible to fully decouple all users in the delay domain. To mitigate this limitation, we propose leveraging frequency-domain characteristics to facilitate user separation. As shown in Figure 2, the energy of each user’s pilot signal is mainly concentrated within its main lobe of width approximately $3N_{d}$ subcarriers [28]. Since the out-of-lobe energy is negligible, it is reasonable to assume that pilot contamination is primarily caused by the two immediate neighboring users on each side. Based on this observation, we subsequently perform user-wise separation in the frequency domain.

We assume that $N_{s}=3N_{d}$ subcarriers per user are involved at the receiver for channel estimation. Considering the symmetry property of the CE filter, the subcarrier set occupied by the *k*-th user can be written as $\Gamma_{k}=\left\{{\langle a_{k}-\frac{N_{s}}{2}+m\rangle }_{N_{c}}\;|\;m=0,1,\ldots ,N_{s}-1\right\}$. For convenience, we further define a injection $M_{k}\left(\cdot \right)$ from $\Gamma_{k}$ to the set $\left\{0,1,\ldots ,N_{s}-1\right\}$, i.e., $M_{k}\left({\langle a_{k}-\frac{N_{s}}{2}+m\rangle }_{N_{c}}\right)=m,\;m\in \left\{0,1,\ldots ,N_{s}-1\right\},\;k\in \left\{0,1,\ldots ,K-1\right\},$ and $M_{k}^{-1}\left(\cdot \right)$ denotes the inverse mapping. Building upon the above assumptions and the received signal model in (15), the decoupled received pilot signal for the *k*-th user is given by

(30) $${\tilde{y}}_{k}={\tilde{X}}_{k}{\tilde{G}}_{k}{\tilde{h}}_{k}+\sum_{u\neq k,u={\langle k-2\rangle }_{K}}^{{\langle k+2\rangle }_{K}}{\tilde{X}}_{u}^{k}{\tilde{G}}_{u}^{k}{\tilde{h}}_{u}^{k}+\tilde{z},$$

where ${\tilde{h}}_{k}={\bar{h}}_{k}\left(M_{k}^{-1}\left(m\right)\right)\in C^{N_{s}\times 1}$ denotes the channel vector of user *k*, and ${\tilde{h}}_{u}^{k}={\bar{h}}_{u}\left(M_{k}^{-1}\left(m\right)\right)\in C^{N_{s}\times 1}$ denotes the channel vector of user *u* restricted to the subband allocated to user *k*. The diagonal matrix ${\tilde{G}}_{k}\in C^{N_{s}\times N_{s}}$ contains the frequency-domain filter coefficients of user *k*, with entries ${\tilde{G}}_{k}\left(m,m\right)=\lambda_{k}\left(M_{k}^{-1}\left(m\right)\right)$. For interfering users, ${\tilde{G}}_{u}^{k}\in C^{N_{s}\times N_{s}}$ denotes the projection of user *u*’s filter onto user *k*’s subband, with entries ${\tilde{G}}_{u}^{k}\left(m,m\right)=\lambda_{u}\left(M_{k}^{-1}\left(m\right)\right)$. Similarly, ${\tilde{X}}_{k}\in C^{N_{s}\times N_{s}}$ and ${\tilde{X}}_{u}^{k}\in C^{N_{s}\times N_{s}}$ are diagonal matrices formed from the equivalent frequency-domain pilot vectors of user *k* and the projection of user *u* onto user *k*’s subband, respectively, with entries ${\tilde{X}}_{k}\left(m,m\right)={\bar{x}}_{k}\left(M_{k}^{-1}\left(m\right)\right)$ and ${\tilde{X}}_{u}^{k}\left(m,m\right)={\bar{x}}_{u}\left(M_{k}^{-1}\left(m\right)\right)$. $\tilde{z}∼\mathrm{CN}\left(0,\sigma_{z}^{2}I_{N_{s}}\right)$ denotes the AWGN vector.

The LS estimate corresponding to (30) can be expressed as

(31) $${\tilde{\xi }}_{k,\mathrm{ls}}=\frac{1}{c}{\tilde{X}}_{k}^{H}{\tilde{y}}_{k}={\tilde{\xi }}_{k}+\sum_{u\neq k,u={\langle k-2\rangle }_{K}}^{{\langle k+2\rangle }_{K}}{\tilde{\Theta }}_{u}^{k}{\tilde{\xi }}_{u}^{k}+\frac{1}{c}{\tilde{X}}_{k}^{H}\tilde{z},$$

where ${\tilde{\xi }}_{k}={\tilde{G}}_{k}{\tilde{h}}_{k}\in C^{N_{s}\times 1}$ represents the equivalent channel of user *k*, ${\tilde{\xi }}_{u}^{k}=j^{\left(u-k\right)}e^{j\pi \frac{\Delta_{k}-\Delta_{u}+2\left(k+1\right)N_{d}}{2N_{d}}}{\tilde{G}}_{u}^{k}{\tilde{h}}_{u}^{k}\in C^{N_{s}\times 1}$ denotes the interfering channel component from user *u*, and ${\tilde{\Theta }}_{u}^{k}$ is a diagonal phase-rotation matrix accounting for the phase offset between users *u* and *k*, defined as

(32) $${\tilde{\Theta }}_{u}^{k}=\mathrm{diag}\left(1,e^{j\frac{3\pi (\Delta_{k}-\Delta_{u})}{N_{s}}},\ldots ,e^{j\frac{3\pi \left(\Delta_{k}-\Delta_{u}\right)\left(N_{s}-1\right)}{N_{s}}}\right)\in C^{N_{s}\times N_{s}},$$

which corresponds to a delay-domain shift of $\frac{3}{2}\left(\Delta_{k}-\Delta_{u}\right)$ samples under the $N_{s}$-point IDFT. Considering that the maximum channel delay spread under the $N_{s}$-point IDFT is limited to $\tilde{L}=\frac{3L}{\Phi }$, interference from adjacent users can be effectively suppressed by ensuring that the frequency-domain shift factors satisfy

(33) $$|\Delta_{k}-\Delta_{u}|>\frac{2L}{\Phi },\;u\in \left\{{\langle k-2\rangle }_{K},{\langle k-1\rangle }_{K},{\langle k+1\rangle }_{K},{\langle k+2\rangle }_{K}\right\}.$$

To satisfy the interference avoidance condition, a feasible construction of the frequency-domain shift factors ${\left\{\Delta_{k}\right\}}_{k=0}^{K-1}$ for *K* users is given as follows. Let K=5Q+R with $Q=\left\lfloor \frac{K}{5}\right\rfloor$ and remainder ${\langle K\rangle }_{5}$. For the main groups (users indexed from 1 to 5Q), the shift factors are assigned periodically as

(34) $$\Delta_{5q+i-1}=i\Delta ,\;q\in \left\{0,1,\ldots ,Q-1\right\},\;i\in \left\{1,2,3,4,5\right\}.$$

The remaining *R* users, indexed from 5Q+1 to 5Q+R, are assigned shift factors by

(35) $$\left\{\Delta_{5Q+1},\ldots ,\Delta_{5Q+R}\right\}=\left\{\begin{array}{cc} \{3\Delta \}, & R=1,\\ \{2\Delta ,3\Delta \}, & R=2,\\ \{2\Delta ,3\Delta ,4\Delta \}, & R=3,\\ \{\Delta ,2\Delta ,3\Delta ,4\Delta \}, & R=4. \end{array}\right.$$

This construction ensures that each of the *K* users satisfies the joint decoupling condition in (33) with its four adjacent users.

**Remark** **1.** *In practical systems, the delay-domain channel length L is generally shorter than the CP length $N_{g}$. Therefore, the inequality $\frac{5L}{\Phi }<\frac{5N_{g}}{\Phi }<\frac{N_{c}}{\Phi }$ typically holds. Consequently, it is straightforward to find a Δ satisfying $\frac{10L}{\Phi }<5\Delta <2N_{d},$ which ensures that the joint decoupling condition in (33) can be achieved according to (35).*

Based on (31) and the pilot sequence constructed using the proposed scheme, the LS channel estimate of the *k*-th user in the delay domain is given by

(36) $${\tilde{\xi }}_{k,\mathrm{dl}}^{\tau }=\tilde{P}W_{N_{s}}^{H}{\tilde{\xi }}_{k,\mathrm{ls}}\approx \tilde{P}W_{N_{s}}^{H}{\tilde{\xi }}_{k}+\frac{1}{c}\tilde{P}W_{N_{s}}^{H}{\tilde{X}}_{k}^{H}\tilde{z}\in C^{(\tilde{L}+1)\times 1},$$

where $\tilde{P}\in R^{\left(\tilde{L}+1\right)\times N_{s}}$ is a selection matrix that extracts the first $\tilde{L}+1$ delay taps. Based on the delay-domain LS estimate ${\tilde{\xi }}_{k,\mathrm{ls}}^{\tau }$, the denoised LS (DN-LS) estimate in the frequency domain is obtained as

(37) $${\tilde{\xi }}_{k,\mathrm{dl}}=W_{N_{s}}{\tilde{P}}^{T}{\tilde{\xi }}_{k,\mathrm{dl}}^{\tau }\approx {\tilde{\xi }}_{k}+\frac{1}{c}W_{N_{s}}{\tilde{P}}^{T}\tilde{P}W_{N_{s}}^{H}{\tilde{X}}_{k}^{H}\tilde{z}\in C^{N_{s}\times 1}.$$

To further enhance the channel estimation performance under low signal-to-noise ratio (SNR) conditions, LMMSE estimation is necessary. Two low-complexity LMMSE estimatiosn schemes, namely DFT-based PDP-eestimation-aided LMMSE channel estimation (DPMCE) and estimation of signal parameters via rotational invariance techniques (ESPRIT)-based PDP-estimation-aided LMMSE channel estimation (EPMCE), were proposed in [28], both of which are developed based on frequency-domain LS estimates. However, in the scenario illustrated below in Figure 1, the frequency-domain LS estimates suffer from strong interference and are thus not directly applicable. This motivates the development of DN-LS-based versions of DPMCE and EPMCE in this work.

To facilitate the LMMSE estimation, the covariance matrix of the DN-LS estimate for the *k*-th user is defined as

(38) $$R_{{\tilde{\xi }}_{k,\mathrm{dl}}}=E\left\{{\tilde{\xi }}_{k,\mathrm{dl}}{\tilde{\xi }}_{k,\mathrm{dl}}^{H}\right\}=R_{{\tilde{\xi }}_{k}}+\sigma_{z}^{2}W_{N_{s}}{\left({\tilde{P}}^{T}\tilde{P}\right)}^{2}W_{N_{s}}^{H}\in C^{N_{s}\times N_{s}},$$

where $R_{{\tilde{\xi }}_{k}}\in C^{N_{s}\times N_{s}}$ denotes the covariance matrix of the equivalent frequency-domain channel for the *k*-th user.

**Remark** **2.** *The matrix $R_{{\tilde{\xi }}_{k,\mathrm{dl}}}$ becomes rank-deficient due to the noise suppression introduced in the time domain, and therefore prevents the direct application of conventional LMMSE estimation.*

**Proof.**  The rank of $R_{{\tilde{\xi }}_{k,\mathrm{dl}}}$ satisfies

(39) $$\begin{array}{cc} \mathrm{rank}\left(R_{{\tilde{\xi }}_{k,\mathrm{dl}}}\right)\; & =\mathrm{rank}\left(W_{N_{s}}^{H}R_{{\tilde{\xi }}_{k,\mathrm{dl}}}W_{N_{s}}\right)\\ \; & \leq \mathrm{rank}\left(W_{N_{s}}^{H}R_{{\tilde{\xi }}_{k}}W_{N_{s}}\right)+\mathrm{rank}\left({\left({\tilde{P}}^{T}\tilde{P}\right)}^{2}\right)\\ \; & \leq 2\left(\tilde{L}+1\right)<N_{s}. \end{array}$$

   □

To overcome this limitation, a regularized LMMSE estimator based on (37) is given by

(40) $${\tilde{\xi }}_{k,\mathrm{lm}}=R_{{\tilde{\xi }}_{k}}{\left(R_{{\tilde{\xi }}_{k}}+\sigma_{z}^{2}\mathrm{diag}\left(W_{N_{s}}{\tilde{P}}^{T}\tilde{P}W_{N_{s}}^{H}+\epsilon I_{N_{s}}\right)\right)}^{-1}{\tilde{\xi }}_{k,\mathrm{dl}}\in C^{N_{s}\times 1},$$

where ϵ is a regularization parameter introduced to approximate the colored noise covariance by a diagonal matrix and to enhance the numerical stability of matrix inversion. Based on this regularized LMMSE estimator, we further develop DN-DPMCE and DN-EPMCE. We first obtain the estimated PDP based on the delay-domain LS estimate as

(41) $${\hat{r}}_{k}^{\tau }=P_{+}\;\left\{{\tilde{P}}^{T}\left({\left|{\tilde{\xi }}_{k,\mathrm{ls}}^{\tau }\right|}^{⊙2}-\sigma_{z}^{2}{\left|\tilde{P}W_{N_{s}}^{H}\right|}^{⊙2}\right)\right\}\in R^{N_{s}\times 1},$$

where $P_{+}\left(\cdot \right)$ denotes an element-wise projection that sets all negative entries to zero. Assuming that the equivalent channel taps in the delay domain are uncorrelated, the corresponding delay-domain covariance matrix is given by

(42) $$R_{k}^{\tau }=\mathrm{diag}\left\{{\hat{r}}_{k}^{\tau }\right\}\in R^{N_{s}\times N_{s}}.$$

The corresponding frequency-domain covariance matrix is then obtained as

(43) $${\hat{R}}_{{\tilde{\xi }}_{k}}=W_{N_{s}}R_{k}^{\tau }W_{N_{s}}^{H}\in C^{N_{s}\times N_{s}}.$$

By substituting this into (40), the DN-DPMCE estimate is given by

(44) $${\tilde{\xi }}_{k,\mathrm{dd}}=W_{N_{s}}R_{k}^{\tau }{\left(R_{k}^{\tau }+\gamma \sigma_{z}^{2}I_{N_{s}}\right)}^{-1}W_{N_{s}}^{H}{\tilde{\xi }}_{k,\mathrm{dl}}\in C^{N_{s}\times 1},$$

where $\gamma =\epsilon +\tilde{L}/N_{s}$. Since all matrices involved in the inversion are diagonal, the DN-DPMCE estimator exhibits low computational complexity. The corresponding channel estimate obtained by removing the filter response from the equivalent estimate is given by ${\tilde{h}}_{k,\mathrm{dd}}={\tilde{G}}_{k}^{-1}{\tilde{\xi }}_{k,\mathrm{dd}}$. Based on ${\tilde{h}}_{k,\mathrm{dd}}$, the PDP parameters ${\left\{{\hat{\beta }}_{k},{\hat{\sigma }}_{\tau ,k,p}^{2},{\hat{\tau }}_{k,p}\right\}}_{p=0}^{P_{k}-1}$ can be further extracted using spatial smoothing and ESPRIT [28]. Based on the estimated PDP parameters and the channel model in (14), the equivalent channel covariance matrix can be reconstructed as

(45) $$R_{{\tilde{\xi }}_{k}}^{\mathrm{pdp}}=\sum_{p=1}^{P_{k}}{\hat{\beta }}_{k}{\hat{\sigma }}_{\tau ,k,p}^{2}{\tilde{G}}_{k}b_{N_{s}}\left({\hat{\tau }}_{k,p}\right)b_{N_{s}}^{H}\left({\hat{\tau }}_{k,p}\right){\tilde{G}}_{k}^{H}=A_{k}\Sigma_{k}A_{k}^{H}\in C^{N_{s}\times N_{s}},$$

where $\Sigma_{k}={\hat{\beta }}_{k}\mathrm{diag}\left(\left[{\hat{\sigma }}_{\tau ,k,1}^{2},{\hat{\sigma }}_{\tau ,k,2}^{2},\ldots ,{\hat{\sigma }}_{\tau ,k,P_{k}}^{2}\right]\right)\in R^{P_{k}\times P_{k}}$ denotes the estimated power of each path, $A_{k}={\tilde{G}}_{k}B_{N_{s}}\in C^{N_{s}\times P_{k}}$. Here, the delay-domain steering matrix $B_{N_{s}}$ is defined as

(46) $$B_{N_{s}}=\left[b_{N_{s}}\left({\hat{\tau }}_{k,1}\right),b_{N_{s}}\left({\hat{\tau }}_{k,2}\right),\ldots ,b_{N_{s}}\left({\hat{\tau }}_{k,P_{k}}\right)\right]\in C^{N_{s}\times P_{k}}.$$

Substituting (45) into (40) and applying the Woodbury matrix identity, the DN-EPMCE estimate ${\tilde{\xi }}_{k,\mathrm{de}}$ is obtained as

(47) $$\begin{array}{cc} {\tilde{\xi }}_{k,\mathrm{de}} & =A_{k}\Sigma_{k}A_{k}^{H}{\left(A_{k}\Sigma_{k}A_{k}^{H}+\gamma \sigma_{z}^{2}I_{N_{s}}\right)}^{-1}{\tilde{\xi }}_{k,\mathrm{dl}}\\ & =\frac{1}{\gamma \sigma_{z}^{2}}A_{k}{\left(\frac{1}{\gamma \sigma_{z}^{2}}A_{k}^{H}A_{k}+\Sigma_{k}^{-1}\right)}^{-1}A_{k}^{H}{\tilde{\xi }}_{k,\mathrm{dl}}\in C^{N_{s}\times 1}. \end{array}$$

The matrix inversion complexity in (47) is $O({P_{k}}^{3})$, which is computationally feasible in scenarios with a limited number of multipaths, such as satellite communications. Moreover, in DN-EPMCE, the PDP parameters are obtained via the truncated SVD procedure in [28], whose complexity is $O(P_{k}N_{d}^{2})$ since only the dominant $P_{k}$ components are retained. As the PDP is a slow-varying second-order statistic that evolves much more slowly than the instantaneous channel coefficients, it does not require frequent updates and can be reused across multiple coherence intervals. Consequently, the associated overhead and latency are relatively small in practical operating conditions.

## 4. Low-Complexity Equalizer Design

Although the proposed CE-CP-OFDMA scheme offers improved spectral efficiency, its performance is susceptible to MAI and ISI, resulting from frequency band overlap and multipath propagation. To eliminate both MAI and ISI, it is necessary to design an equalizer specifically for the proposed CE-CP-OFDMA scheme. Therefore, in this section, we first establish a simplified received signal model for equalization and then develop a frequency-domain LMMSE equalizer for the proposed CE-CP-OFDMA scheme.

### 4.1. Simplified Received Signal Model for Equalization

As illustrated below in Figure 1, in the proposed CE-CP-OFDMA scheme, each user is assigned $N_{d}$ subcarriers for data transmission. Accordingly, the received signal on the corresponding $N_{d}$ subcarriers are utilized for equalization. To establish the simplified received signal model for equalization, we first distinguish the desired signal components from the interference among different users. By combining (10) and (15), the received signal within the *u*-th user’s frequency band $y_{u,l}\in C^{N_{d}\times 1}$ can be expressed as

(48) $$y_{u,l}=H_{u,l}G_{u}s_{u,l}+y_{u,l}^{i}+z_{u,l},$$

where $y_{u,l}^{i}$ denotes the interference term, given by

(49) $$y_{u,l}^{i}=\left\{\begin{array}{cc} \sum_{k\neq u,\;k=2i+1}^{K-1}H_{k,l}^{u}G_{k}^{u}J_{N_{d}}s_{k,l}^{*}+\sum_{k\neq u,\;k=2i}^{K-1}H_{k,l}^{u}G_{k}^{u}s_{k,l},\; & \mathrm{if}\;u\;\mathrm{is}\;\mathrm{even},\\ \sum_{k\neq u,\;k=2i}^{K-1}H_{k,l}^{u}G_{k}^{u}J_{N_{d}}s_{k,l}^{*}+\sum_{k\neq u,\;k=2i+1}^{K-1}H_{k,l}^{u}G_{k}^{u}s_{k,l},\; & \mathrm{if}\;u\;\mathrm{is}\;\mathrm{odd}. \end{array}\right.$$

In the above, $i\in \{0,1,\cdots ,\left\lfloor \frac{K-1}{2}\right\rfloor \}$, $y_{u,l}\left(m\right)=y_{l}\left(M_{u}^{-1}\left(m\right)\right)$. $H_{u,l}$ is the channel matrix of the *u*-th user and its *m*-th diagonal element $H_{u,l}\left(m,m\right)=h_{u,l}\left(M_{u}^{-1}\left(m\right)\right)$, while $H_{u,l}^{k}$ is the *u*-th user’s channel matrix in the range of the *k*-th user’s frequency band, with the *m*-th diagonal element $H_{u,l}^{k}\left(m,m\right)\;=\;h_{u,l}\left(M_{k}^{-1}\left(m\right)\right)$, $G_{u}$ is the filter coefficient matrix in the frequency band occupied by the *u*-th user with $G_{u}\left(m,m\right)=\lambda_{u}^{T}\left(M_{u}^{-1}\left(m\right)\right)$, and its filter coefficient matrix in the range of the *k*-th user is given by $G_{u}^{k}\left(m,m\right)=\lambda_{u}^{T}\left(M_{k}^{-1}\left(m\right)\right)$.

According to (48) and (49), the frequency-domain received signal $y_{u,l}$ within the *u*-th user’s frequency band contains both the desired frequency-domain data vector $s_{u,l}$ of the *u*-th user and those of the interfering users. Therefore, it is necessary to perform joint equalization to eliminate the MAI. Since $y_{u,l}$ contains both $s_{k,l}$ and $s_{k,l}^{*}$, a preprocessing step is required to facilitate the establishment of the joint received signal model for all *K* users. For simplicity, the number of users *K* is assumed to be even in the following derivations. Specifically, we stack the received signal $y_{u,l}$ for even *u* and $y_{u,l}^{*}$ for odd *u*, and the new received signal vector is

(50) $${\bar{y}}_{l}={\left[{\left(y_{0,l}\right)}^{T},{\left(y_{1,l}^{*}\right)}^{T},\cdots ,{\left(y_{K-2,l}\right)}^{T},{\left(y_{K-1,l}^{*}\right)}^{T}\right]}^{T}\in C^{KN_{d}\times 1},$$

correspondingly, stack $s_{u,l}$ for even *u* and $s_{u,l}^{*}$ for odd *u*, i.e.,

(51) $${\bar{s}}_{l}={\left[{\left(s_{0,l}\right)}^{T},{\left(s_{1,l}^{*}\right)}^{T},\cdots ,{\left(s_{K-2,l}\right)}^{T},{\left(s_{K-1,l}^{*}\right)}^{T}\right]}^{T}\in C^{KN_{d}\times 1}.$$

In the subsequent equalization, we apply the preprocessed received signal ${\bar{y}}_{l}$ as the observation instead of $y_{l}$. Therefore, combining (48) and the structure of ${\bar{y}}_{l}$, we can obtain the following linear expression as

(52) $${\bar{y}}_{l}={\bar{A}}_{l}{\bar{s}}_{l}+{\bar{z}}_{l},$$

where ${\bar{z}}_{l}={\left[{\left(z_{0,l}\right)}^{T},{\left(z_{1,l}^{*}\right)}^{T},\cdots ,{\left(z_{K-1,l}^{*}\right)}^{T}\right]}^{T}$. According to the subcarrier allocation methods investigated in Section 2.2, when adjacent users have a partial overlap of subcarriers, the matrix ${\bar{A}}_{l}$ can be defined as

(53) $${\bar{A}}_{l}=\left[\begin{array}{cccc} A_{0,0} & A_{0,1} & \ldots & A_{0,K-1}\\ A_{1,0}^{*} & A_{1,1}^{*} & \ldots & A_{1,K-1}^{*}\\ \vdots & \vdots & \ddots & \vdots \\ A_{K-2,0} & A_{K-2,1} & \ldots & A_{K-2,K-1}\\ A_{K-1,0}^{*} & A_{K-1,1}^{*} & \ldots & A_{K-1,K-1}^{*} \end{array}\right]\in C^{KN_{d}\times KN_{d}},$$

where each block $A_{u,k}\in C^{N_{d}\times N_{d}}$ represents the coupling between the *u*-th and *k*-th users in the frequency domain and follows the rule

(54) $$A_{u,k}=\left\{\begin{array}{cc} H_{k,l}^{u}G_{k}^{u}, & \mathrm{if}\;(u,k)\;\mathrm{have}\;\mathrm{the}\;\mathrm{same}\;\mathrm{parity},\\ H_{k,l}^{u}G_{k}^{u}J_{N_{d}}, & \mathrm{if}\;(u,k)\;\mathrm{have}\;\mathrm{different}\;\mathrm{parities}. \end{array}\right.$$

**Remark** **3.** *When the system allocates exclusive subcarriers for each user, the MAI from adjacent users is negligible, so the non-diagonal block matrices in ${\bar{A}}_{l}$ are reduced to the all-zero ones, i.e.,*

(55) $${\bar{A}}_{l}=\mathrm{diag}\left(\left[A_{0,0},{\left(A_{1,1}\right)}^{*},\ldots ,{\left(A_{K-1,K-1}\right)}^{*}\right]\right).$$

Because of the optimized out-of-band energy of the filter, the impact of MAI caused by the users whose subcarriers are far away from the current user can be negligible. Hence, without loss of generality, we only consider the MAI caused by two users left and right adjacent to the current user. Take the *u*-th user as an example, the received signal $y_{u,l}$ can be simplified to

(56) $$y_{u,l}\approx H_{u,l}G_{u}s_{u,l}+\sum_{i={\langle u+1\rangle }_{K},{\langle u-1\rangle }_{K}}H_{i,l}^{u}G_{i}^{u}J_{N_{d}}s_{i,l}^{*}+\sum_{i={\langle u+2\rangle }_{K},{\langle u-2\rangle }_{K}}H_{j,l}^{u}G_{j}^{u}s_{j,l}+z_{u,l},$$

where $H_{u,l}G_{u}s_{u,l}$ respresents the *u*-th user’s received signal without interference and noise, while the remaining four terms at the right-hand side of (56) represent the main MAI. Accordingly, we define a simplified version of the matrix ${\bar{A}}_{l}$, denoted as ${\bar{A}}_{l}^{\prime }$, where $A_{u,k}=0_{N_{d}\times N_{d}}$ for all $k\notin \{{\langle u-2\rangle }_{K},{\langle u-1\rangle }_{K},{\langle u\rangle }_{K},{\langle u+1\rangle }_{K},{\langle u+2\rangle }_{K}\}$.

### 4.2. Frequency Domain LMMSE Equalization

As is well known, LMMSE equalization can effectively mitigate the MAI and ISI while maintaining relatively low implementation complexity in practice [27,32]. Assuming that the data vector $d_{l}$ consists of independent and identically distributed (i.i.d.) elements, the FD-LMMSE estimate ${\bar{s}}_{l}^{\prime \prime }$ can be expressed based on (52), (53) and (56) as

(57) $${{\bar{s}}^{\prime \prime }}_{l}\left(m\right)=e_{m}^{T}{\left({\left({\bar{A}}_{l}^{\prime }\right)}^{H}{\bar{A}}_{l}^{\prime }V_{l}+\sigma_{z}^{2}I_{N_{c}}\right)}^{-1}{\left({\bar{A}}_{l}^{\prime }\right)}^{H}\left({\bar{y}}_{l}-{\bar{A}}_{l}^{\prime }\mu_{l}\right)+\mu_{l}\left(m\right),$$

where $V_{l}=\mathrm{cov}\left(d_{l},d_{l}\right)$ is a diagonal matrix. $\mu_{l}={\left[{\left(\mu_{0,l}\right)}^{T},{\left(\mu_{1,l}^{*}\right)}^{T},\ldots ,{\left(\mu_{K-1,l}^{*}\right)}^{T}\right]}^{T}\in C^{KN_{d}\times 1}$ denotes the frequency-domain expectation vector, where $\mu_{u,l}={\left[W_{2N_{d,u}}E\left\{d_{u,l}\right\}\right]}_{1:N_{d}}$ denotes the front half part of the transformed expectation vector. $E\left\{d_{u,l}\right\}$ is the expectation of the detection values fed back by the decoder. To further improve detection performance, the LMMSE estimator is extended to the turbo iterative version, where the signal detection incorporates additional information from previous iterations. The updated detection process is expressed as

(58) $${{\bar{s}}^{\prime }}_{l}\left(m\right)=e_{m}^{T}{\left({\left({\bar{A}}_{l}^{\prime }\right)}^{H}{\bar{A}}_{l}^{\prime }V_{l}+\sigma_{z}^{2}I_{N_{c}}\right)}^{-1}{\left({\bar{A}}_{l}^{\prime }\right)}^{H}\left({\bar{y}}_{l}-{\bar{A}}_{l}^{\prime }\mu_{l}\right)+\Omega_{m}\mu_{l}\left(m\right),$$

with

(59) $$\Omega_{m}=e_{m}^{T}{\left({\left({\bar{A}}_{l}^{\prime }\right)}^{H}{\bar{A}}_{l}^{\prime }V_{l}+\sigma_{z}^{2}I_{N_{c}}\right)}^{-1}{\left({\bar{A}}_{l}^{\prime }\right)}^{H}{\bar{A}}_{l}^{\prime }e_{m}.$$

Then, the *u*-th user’s detected frequency domain signal ${\hat{s}}_{u,l}$ can be extracted from ${{\bar{s}}^{\prime }}_{l}$ according to (51), and ${\hat{q}}_{u,l}$ can also be obtained.

It can be observed that in the computation of the proposed receiver, both (58) and (59) involve high-dimensional $KN_{d}\times KN_{d}$ matrix inversions and matrix multiplications, which lead to high computational complexity and potentially large power consumption. However, the $KN_{d}$-dimensional matrices consist entirely of $N_{d}\times N_{d}$ diagonal or anti-diagonal blocks. This structure enables the use of a block-wise recursive Schur complement method [33] to avoid direct inversion of large matrices. In particular, during the recursive block-wise Schur complement inversion, the required multiplications between two $KN_{d}\times KN_{d}$ matrices are performed at the block level. Each product of two $N_{d}\times N_{d}$ diagonal or anti-diagonal blocks costs only $O(N_{d})$ operations, instead of $O(N_{d}^{3})$ for dense blocks. Since a multiplication of two K×K block matrices involves on the order of $K^{3}$ such block products, the overall complexity of each large matrix multiplication is reduced from $O\left(K^{3}N_{d}^{3}\right)=O\left({\left(KN_{d}\right)}^{3}\right)$ to $O(K^{3}N_{d})$. Moreover, all final-stage inversions are performed only on $N_{d}\times N_{d}$ diagonal or anti-diagonal matrices, each requiring $O(N_{d})$ operations. Since the recursive Schur complement procedure contains approximately $\log_{2}K-1$ stages, the total complexity of the proposed receiver scales as $O(K^{3}N_{d}\log_{2}K),$ which significantly reduces the computational burden compared with a direct dense-matrix implementation.

After FD-LMMSE equalization, several post-processing operations are performed to obtain the final symbol estimate ${{\bar{d}}^{\prime }}_{u,l}$. Similar to the transmitter discussed in Section 2, we introduce the frequency-domain postprocessing here to implement a compatible implementation structure of the receiver, which is defined by

(60) $${{\bar{q}}^{\prime }}_{u,l}=\frac{1}{2\sqrt{2}}\left[\begin{array}{cc} I_{N_{d,u}} & je^{j\pi /(2N_{d})}\Phi_{N_{d,u}}^{*} \end{array}\right]\left[\begin{array}{cc} I_{N_{d,u}} & I_{N_{d,u}}\\ I_{N_{d,u}} & -I_{N_{d,u}} \end{array}\right]\left({\hat{q}}_{u,l}+J_{2N_{d,u}}{\left({\hat{q}}_{u,l}\right)}^{*}\right)\in C^{N_{d}\times 1}.$$

After the above postprocessing, the $N_{d,u}$-point GDFT coefficient vector ${{\bar{q}}^{\prime }}_{u,l}$ can be obtained, which is aligned with the following $N_{d,u}$-point IDFT in conventional DFT-s-OFDM receiver. With the operation of the phase rotation given by $\Phi_{N_{d,u}}^{H}$ and $N_{d,u}$-point IDFT, the recovered symbol vector ${{\bar{d}}^{\prime }}_{u,l}$ of the *u*-th user in the *l*-th block is

(61) $${{\bar{d}}^{\prime }}_{u,l}=\Omega_{m}^{-1}W_{N_{d,u}}^{H}\Phi_{N_{d,u}}^{H}{{\bar{q}}^{\prime }}_{u,l}.$$

Overall, Figure 2 illustrates the receiver structure of the proposed approach, which is fully compatible with the CP-OFDMA framework. The corresponding reception algorithm is summarized in Algorithm 2. It is worth emphasizing that, except for the joint equalization block, all processing components in Figure 2 exhibit essentially the same computational complexity as their counterparts in [28]. The primary distinction arises in the equalization stage. In [28], the equalizer has a complexity of $O(KN_{d})$, whereas the proposed joint equalizer increases this complexity to $O(K^{3}N_{d}\log_{2}K)$. Nevertheless, considering that the number of users *K* is typically much smaller than the number of occupied subcarriers $N_{d}$ in practical systems, the additional computational burden introduced by the joint equalizer remains limited, while providing a significant improvement in spectral efficiency compared with the CE scheme in [28].
**Algorithm 2** Receiver Algorithm for the Proposed CE-CP-OFDM System**Input:** Frequency-domain received signal $y\in C^{{\bar{N}}_{c}\times 1}$% Channel estimation based on the received pilot signal1:**for** k=0 to K−1 **do**2:    Perform frequency-domain decoupling to obtain ${\tilde{y}}_{k}$.3:    Compute ${\tilde{\xi }}_{k,\mathrm{ls}}$ via (31).4:    Compute ${\tilde{\xi }}_{k,\mathrm{dl}}^{\tau }$ via (36) to achieve further decoupling in the delay domain.5:    Compute ${\tilde{\xi }}_{k,\mathrm{dl}}$ via (37).6:    Compute ${\tilde{\xi }}_{k,\mathrm{dd}}$ via (41)–(44).7:    Compute ${\tilde{h}}_{k,\mathrm{dd}}={\tilde{G}}_{k}^{-1}{\tilde{\xi }}_{k,\mathrm{dd}}$.8:    Compute ${\left\{{\hat{\beta }}_{k},{\hat{\sigma }}_{\tau ,k,p}^{2},{\hat{\tau }}_{k,p}\right\}}_{p=1}^{P_{k}}$ from ${\tilde{h}}_{k,\mathrm{dd}}$ using spatial smoothing and ESPRIT.9:    Compute ${\tilde{\xi }}_{k,\mathrm{de}}$ via (45)–(47).10:**end for**% Equalization based on the received data signal11:Compute ${\bar{y}}_{l}$, ${\bar{s}}_{l}$ via (50) and (51).12:Compute ${\bar{s}}_{l}^{\prime }$ via (58).13:**for** u=0 to K−1 **do**14:    Compute ${{\bar{q}}^{\prime }}_{u,l}$ via (60)15:    Compute ${{\bar{d}}^{\prime }}_{u,l}$ via (61).16:**end for****Output:** ${{\bar{d}}^{\prime }}_{u,l}\in C^{N_{d}\times 1}$.

## 5. Simulation Results

This section evaluates the receiver performance of the proposed CE-CP-OFDMA scheme in terms of channel estimation accuracy and BER. A more realistic multiuser scenario with heterogeneous link qualities is considered in order to rigorously assess the robustness of the proposed interference mitigation techniques. Specifically, two extreme but representative interference conditions are examined. In the first condition, the target user and the interfering users occupying the adjacent frequency subbands experience comparable link qualities and therefore have approximately the same received signal power. Under this setting, the signal-to-interference ratio (SIR) within the target user’s allocated subband is about 2 dB, representing a weak-interference scenario. In the second condition, the target user has a significantly weaker link quality, whereas the adjacent-subband interferers possess much stronger link qualities. To model this power imbalance, these interferers are assumed to have ten times higher received power than the target user, resulting in an SIR of approximately −8 dB within the target user’s subband. These two contrasting interference scenarios enable a comprehensive evaluation of the robustness of the proposed joint channel-estimation and joint equalization scheme under practical variations in inter-user power disparities.

In the simulations, identical transmission parameters are assumed for all users unless otherwise specified, where each user is allocated $N_{d}$ subcarriers for transmitting $N_{d}$ complex data symbols per OFDM symbol. The key simulation parameters are summarized in Table 1.

### 5.1. PAPR Performance

**Figure 3:**
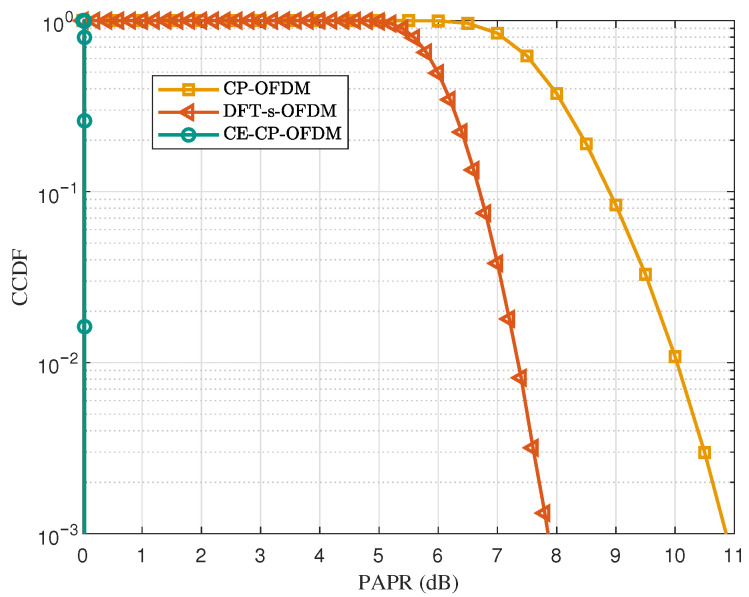
PAPR comparison among CE-CP-OFDMA, CP-OFDM, and DFT-s-OFDM waveforms.

Figure 3 illustrates the PAPR performance of the CP-OFDM, DFT-s-OFDM, and CE-CP-OFDM waveforms. It can be clearly observed that the proposed CE-CP-OFDM waveform achieves a remarkable reduction in PAPR. Taking the CCDF level of $10^{-3}$ as a reference, CE-CP-OFDM attains an ultra-low PAPR of 0 dB owing to its CE property, while DFT-s-OFDM and CP-OFDM exhibit PAPR values of approximately 7.9 dB and 10.8 dB, respectively. This substantial PAPR advantage allows CE-CP-OFDM to operate with a much smaller output back-off when passing through the PA, thereby significantly improving power efficiency under nonlinear distortion constraints.

### 5.2. Channel Estimation Performance

For the evaluation of channel estimation performance, we consider the NTN tapped delay line channel with delay profile D (NTN-TDL-D) specified in 3GPP TR 38.811 [34], which serves as a representative frequency-selective fading model for NTN scenarios. Following Table 6.7.2-1b of 3GPP TR 38.811, the delay spread is configured as 37 ns to capture typical propagation characteristics in NTN environments. To quantify the estimation accuracy, the normalized mean square error (NMSE) is employed, defined as

(62) $$\mathrm{NMSE}=10\log_{10}\left(\frac{∥\hat{\xi }{-\xi ∥}_{2}^{2}}{{∥\xi ∥}_{2}^{2}}\right),$$

where ξ and $\hat{\xi }$ denote the actual and estimated equivalent channels, respectively. To demonstrate the superiority of the proposed algorithm, we compare it against the following baselines:

- **LS**: The conventional LS channel estimation method, as expressed in (31).
- **DN-LS**: This algorithm first estimates the frequency-domain channels using the LS method and then denoises the estimated channel in the delay domain, as given in (37).
- **DN-DPMCE**: This algorithm converts the LS estimate into the DFT domain for the DFT-domain correlation matrix, from which the frequency-domain correlation matrix is derived. A low-complexity LMMSE estimator is then applied, as expressed in (44).
- **Ideal MMSE**: This serves as the theoretical upper bound for channel estimation performance. It assumes a single-user, interference-free scenario and utilizes the ideal channel correlation matrix to perform MMSE estimation.

**Figure 4:**
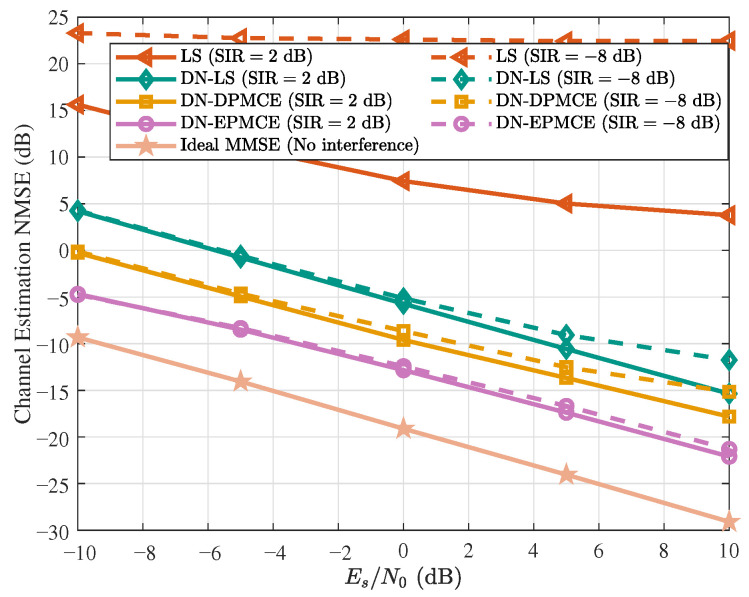
Channel estimation performance under different estimation methods with SIR = 2dB (solid lines) and SIR = −8dB (dashed lines).

To evaluate the channel estimation performance, the proposed method is tested under the two interference scenarios described earlier, namely the weak-interference case (SIR = 2 dB) and the strong-interference case (SIR = −8 dB). The corresponding results are shown in Figure 4. As shown in the figure, the estimation accuracy of all algorithms improves steadily with increasing $E_{s}/N_{0}$. Across the entire SNR range, the proposed DN-EPMCE algorithm consistently achieves the lowest NMSE, demonstrating its superior estimation capability. Compared with DN-DPMCE, DN-EPMCE attains about a 4.5 dB NMSE gain at $E_{s}/N_{0}=0$ dB, owing to its ability to mitigate energy leakage during PDP estimation and thus reconstruct the channel more accurately. DN-DPMCE itself outperforms DN-LS by approximately 4 dB, since its LMMSE-based denoising further suppresses residual noise within the delay-domain window, while DN-LS merely removes the out-of-window noise components. In contrast, the conventional LS estimator exhibits the poorest performance in multiuser interference scenarios, with its accuracy rapidly degrading as the interference level increases. By jointly decoupling signals in both the delay and frequency domains, the proposed DN-EPMCE effectively separates multiuser components and maintains stable performance under different interference conditions. For instance, at $E_{s}/N_{0}=0$ dB, DN-EPMCE achieves an NMSE of approximately −13 dB under both weak (SIR=2 dB) and strong (SIR=−8 dB) interference, with only about 6.5 dB gap relative to the IdealMMSE benchmark, verifying its effectiveness and robustness.

### 5.3. BER Performance

**Figure 5:**
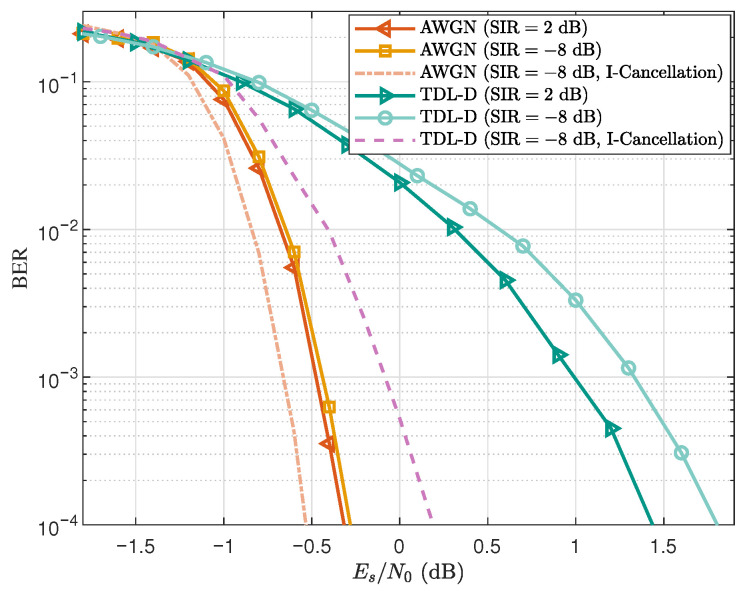
BER performance of the proposed CE-CP-OFDMA scheme under different SIR levels in both AWGN and TDL-D channels.

In terms of BER performance, the robustness of the proposed joint equalization scheme is evaluated under the two interference conditions introduced earlier. Figure 5 presents the BER curves of the proposed CE-CP-OFDMA scheme under these two SIR levels for both AWGN and NTN-TDL-D channels. In the figure, the curve labeled ‘I-Cancellation’ corresponds to the strong-interference case (SIR=−8dB) with an additional interference cancellation step applied after joint equalization. In this scenario, the interfering user exhibits a significantly higher received power than the target user. Owing to the effectiveness of the joint equalization, the interfering signal can be accurately reconstructed and subsequently subtracted from the received signal, after which a low-complexity single-tap equalizer is applied to obtain a more reliable detection result.

From Figure 5, it can be observed that the BER curves corresponding to SIR=2dB and SIR=−8dB almost overlap in the AWGN channel, showing that the interference level has little impact under such ideal conditions. In the NTN-TDL-D frequency-selective channel, the SIR=−8dB case exhibits only about 0.3dB degradation at a BER of $10^{-4}$ compared with the SIR=2dB case. These results indicate that the proposed scheme maintains stable BER performance even under significant power imbalance, demonstrating its strong robustness. Moreover, in the strong-interference scenario with SIR=−8dB, the simulation results show that a significant improvement in BER performance can be achieved by simply appending a low-complexity single-tap equalization module after the joint equalizer. Considering that the SIR=2dB setting naturally represents a more balanced and fair multiuser condition, and for convenience, the subsequent BER simulations are presented only for the SIR=2dB configuration.

**Figure 6:**
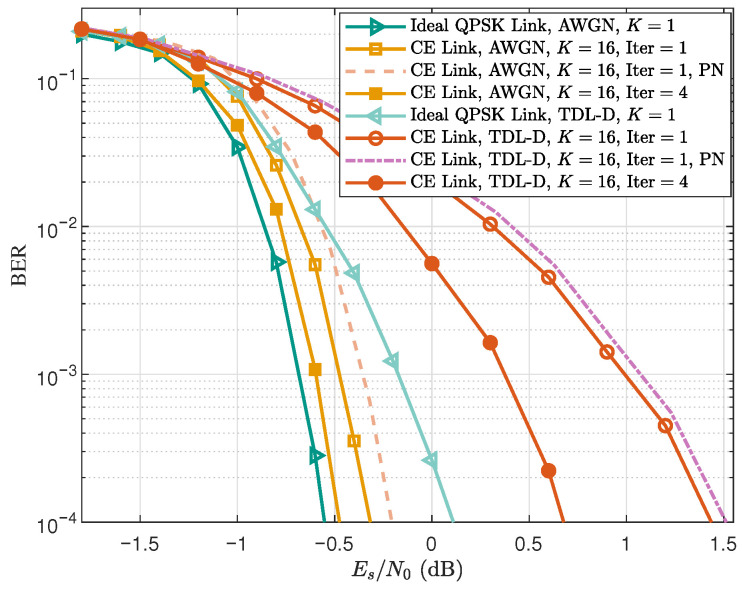
BER performance of the proposed CE-CP-OFDMA scheme in the NTN-TDL-D channel. Dashed curves denote the results with phase noise.

Figure 6 illustrates the BER performance of the proposed CE-CP-OFDMA scheme under both AWGN and NTN-TDL-D channels. The notations ‘Iter =1’ and ‘Iter =4’ correspond to one and four Turbo decoding iterations, respectively, whereas ‘K=1’ and ‘K=16’ denote the cases of single-user and sixteen-user transmission. In addition, ‘CE Link’ and ‘Ideal QPSK Link’ refer to the proposed CE transmission link and the ideal QPSK-modulated OFDM link, respectively. It can be observed that in the AWGN channel, the proposed joint equalization scheme achieves nearly ideal performance, with only 0.3 dB and 0.1 dB gaps at a BER of $10^{-4}$ for ‘Iter =1’ and ‘Iter =4’, respectively. Under the NTN-TDL-D channel, the corresponding gaps increase to 1.5 dB and 0.5 dB, primarily due to frequency-selective fading reducing the interference suppression capability of the joint equalization. Nevertheless, performing four Turbo iterations significantly mitigates this gap: iterative exchange of soft information between equalizer and decoder refines symbol estimates and better suppresses residual interference, thereby improving BER performance under frequency-selective fading conditions.

In addition, we evaluate the impact of oscillator phase noise, as CE waveforms are often employed in high-frequency systems where phase noise can be a critical impairment. Accordingly, two BER curves incorporating phase-noise effects are included in Figure 6. The phase-noise model follows the Ka-band DVB-S2 specification, with parameters taken from Table M.3 of the ETSI EN 302 307-1 V1.4.1 standard [35]. As shown in the figure, the BER degradation caused by phase noise is very small and can be considered negligible in both AWGN and NTN-TDL-D channels. Therefore, under the phase-noise conditions specified in the DVB-S2 standard [35], the proposed CE-CP-OFDMA scheme exhibits good robustness.

**Figure 7:**
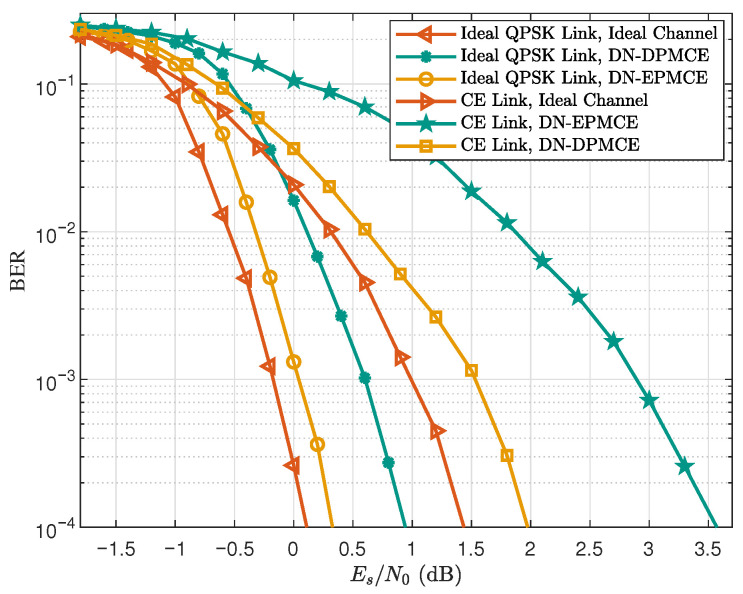
BER performance of different links using different channel estimation methods in the NTN-TDL-D channel (‘K=16’, ‘Iter =1’).

Figure 7 presents the BER performance of different links using different channel estimation methods in the NTN-TDL-D channel. For the Ideal QPSK Link, when targeting a BER of $10^{-4}$, using DN-DPMCE and DN-EPMCE results in approximately 0.3 dB and 0.9 dB degradation, respectively, compared with the ideal-channel case. For the CE Link, the corresponding degradations increase to about 0.5 dB and 1.9 dB. These results show that the CE Link is more sensitive to channel estimation accuracy than the Ideal QPSK Link. The reason is that the joint equalization in the CE Link relies heavily on accurate channel estimates to suppress multiuser interference, so residual estimation errors cause a larger performance loss compared with the ideal OFDM link without multiuser interference.

**Figure 8:**
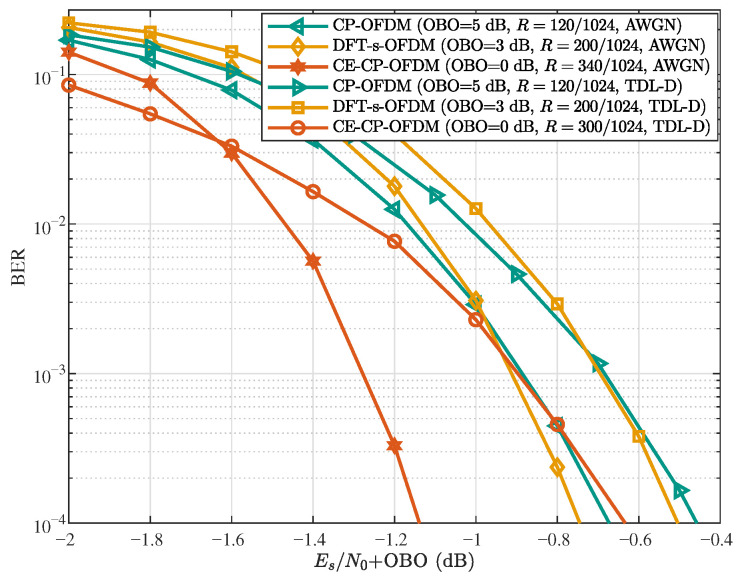
BER performance comparison of CE-CP-OFDM, DFT-s-OFDM, and CP-OFDM under power back-off (‘K=16’, ‘Iter =1’).

To highlight the advantages of the proposed CE-CP-OFDMA scheme, we compare it with DFT-s-OFDM and CP-OFDM, all employing the same subcarrier allocation scheme. Figure 8 presents the BER performance of these three schemes in both AWGN and NTN-TDL-D channels, where the different PA OBO levels arise from their respective waveforms under the same average transmit power. The power back-off levels follow the specifications in 3GPP TS 38.101-2 [36], under which CE-CP-OFDM yields approximately 3 dB and 5 dB higher received $E_{s}/N_{0}$ than DFT-s-OFDM and CP-OFDM for the same transmit power. As illustrated in Figure 8, with a target BER of $10^{-4}$ and comparable decoding performance, CE-CP-OFDM achieves about 2.8 and 1.7 times higher bit rates than CP-OFDM and DFT-s-OFDM, respectively, in the AWGN channel. These gains remain substantial at approximately 2.5 and 1.5 times in the NTN-TDL-D channel, despite slight degradation caused by frequency selectivity. Since all schemes operate over the same bandwidth, these bit-rate improvements directly translate into corresponding gains in spectral efficiency. These results confirm that the proposed CE-CP-OFDMA scheme can substantially improve spectral efficiency, compared with conventional waveforms, when PA OBO is taken into account.

## 6. Conclusions

In this paper, we proposed a CE-CP-OFDM transceiver under the CP-OFDMA framework that achieved high spectral efficiency and low PAPR. We introduced a spectrally efficient subcarrier mapping with partial frequency overlap and established a multiuser received signal model under frequency-selective fading channels. Based on the signal model, an optimal multiuser CE pilot design was developed to minimize joint channel estimation error, and a joint delay–frequency-domain channel estimation method combined with a low-complexity LMMSE estimator was proposed for large-scale multiuser scenarios. To combat ISI and MAI, we established a joint multiuser received-signal model for equalization and designed an iterative FD-LMMSE receiver based on this model. Numerical results demonstrate that the proposed transceiver outperforms conventional waveforms in spectral efficiency in practical scenarios, confirming its effectiveness for spectrum- and power-constrained 6G applications.

In future work, the proposed CE-CP-OFDMA framework may be extended to multi-antenna systems to exploit spatial multiplexing and diversity gains. Another promising direction is the study of asynchronous uplink transmission, where timing misalignment among users may introduce additional multiuser interference and affect the performance of the joint equalizer and channel estimator. In addition, addressing practical implementation challenges—such as the real-time complexity of channel estimation and iterative equalization, and the increasingly severe oscillator phase noise observed in high-frequency systems (e.g., THz)—remains essential. A more detailed analysis of the proposed scheme under strong phase-noise conditions, together with effective mitigation strategies, will be particularly important for enabling efficient deployment of the proposed CE-CP-OFDMA system in future 6G networks.

## Figures and Tables

**Table 1:** Simulation Parameters.

Parameter	Value
Channel model	AWGN and NTN-TDL-D [34]
Carrier frequency	30 GHz
LDPC code rate	379/1024
Modulation scheme	QPSK
Maximum number of users	K=16
Number of signal samples per block	$N_{d}=256$
Number of subcarriers	$N_{c}=4096$
Subcarrier spacing Δf	120 kHz

## Data Availability

Data are contained within the article.
